# Comparing the Results of Written Testing for Advanced Cardiac Life Support Teaching Using Team-based Learning and the “Flipped Classroom” Strategy

**DOI:** 10.7759/cureus.2574

**Published:** 2018-05-03

**Authors:** Mark I Langdorf, Craig L Anderson, Roman E Navarro, Suzanne Strom, C. Eric McCoy, Julie Youm, Mary F Ypma-Wong

**Affiliations:** 1 Department of Emergency Medicine, University of California, Irvine, Irvine, USA; 2 Department of Anesthesiology and Perioperative Care, University of California, Irvine, Irvine, USA; 3 Medical Education, Univeristy of California, Irvine School of Medicine, Irvine, USA; 4 Graduate Division, University of California, Irvine, Irvine , USA

**Keywords:** acls, team-based learning, flipped classroom, medical education

## Abstract

Objectives

We sought to further determine whether cognitive test results changed for advanced cardiac life support (ACLS) taught in the team-based learning/flipped classroom format (TBL/FC) versus a lecture-based (LB) control.

Methods

We delivered 2010 ACLS to two classes of fourth-year medical students in the TBL/FC format (2015–2016), compared to three classes in the LB format (2012–2014). There were 27.5 hours of instruction for the TBL/FC model (TBL - 10.5 hours, podcasts - nine hours, small-group simulation - eight hours), and 20 hours (lectures - 12 hours, simulation - eight hours) in LB. We taught TBL for 13 cardiac cases while LB had none. Didactic content and seven simulated cases were the same in lecture (2012–2014) or in podcast formats (2015–2016). Testing was the same using 50 multiple-choice (MC) format questions, 20 rhythm-matching questions, and seven fill-in management of simulated cases.

Results

Some 468 students enrolled in the course 259 (55.4%) in the LB format in 2012–2014, and 209 (44.6%) in the TBL/FC format in 2015–2016. The scores for two out of three tests (MC and fill-in) increased with TBL/FC. Combined, median scores increased from 93.5% (IQR 90.6, 95.4) to 95.1% (92.5, 96.8, p = 0.0001). More students did not pass one of three tests with LB versus TBL/FC (24.7% versus 18.2%), and two or three parts of the test (8.1% versus 4.3%, p = 0.01). On the contrary, 77.5% passed all three with TBL/FC versus 67.2% with LB (change 10.3%, 95% CI 2.2%–18.2%).

Conclusion

TBL/FC teaching for ACLS improved written test results compared with the LB format.

## Introduction

The passive transfer of knowledge has been reported to be less effective compared to active learning [1-5]. Active learning requires students to perform activities like reading, writing, and problem-solving with discussion, and promotes synthesis and recall of important material  [6]. TBL in small groups is a form of active learning. TBL uses a “flipped classroom” (FC) model to foster content recall. With FC, students review material before class in the form of assigned readings or video podcasts [6]. Then, students work together in class to solve patient care problems [7]. Finally, an instructor explains difficult content and concepts.

Instruction with simulation has been used in advanced cardiac life support (ACLS) for some 30 years, yet the small-group learning format has been studied sparsely [8-9]. The most recent ACLS course offered by the American Heart Association (AHA) has adopted a “blended learning” approach that also flips the classroom as one of its approved formats. In the blended learning format, students watch digital recordings before lectures in the classroom [10]. This new method of ACLS instruction has not yet been well studied. We published a study comparing a TBL/FC learning model similar to the new AHA format with the lecture-based format for ACLS training. This showed a modest increase in written test scores with TBL/FC and a decreased failure rate [11]. However, other studies have shown that such a model might not be more effective compared to traditional lectures [12-13].

Given these conflicting reports, we expanded upon our initial work [11] here, by evaluating another class of 114 students taught using this TBL/FC model, to gauge the effect of this pedagogical shift in the AHA’s ACLS course format. We postulated  that the increase in written test scores for TBL/FC suggesting enhanced learning would be confirmed with a larger sample of two “after” classes, rather than one [11], as shown by better scores on various forms of written testing, compared with three previous  control classes who were instructed on the same material in the LB format.

## Materials and methods

We studied the senior class of 104 students each year. As the ACLS course is “required,” most students enroll except those graduating from Doctorate of Philosophy, Master of Public Health, and Masters in Business Administration programs, whose senior year is variable. As such, 95 final-year students took the TBL/FC ACLS course in 2015, while 113 enrolled in 2016 (n = 209 total in the TBL/FC group).

We taught the simulation component in eight rooms, each with a cardiac defibrillator with an intrinsic transcutaneous pacemaker on a crash cart and an intubation/airway mannequin with high-fidelity simulation (Laerdahl model, “Sim-Man,” Wappingers Falls, New York), located in a 550 sq m simulation center. The TBL content was delivered in a standard tiered-seating lecture room.

We taught two classes (2015 and 2016) of senior medical students the 2010 version of AHA ACLS in a TBL/FC format, and compared written test scores to three control classes taught in the traditional LB format (2012, 2013, and 2014). Table 1 shows that the TBL/FC format had a total of 27.5 hours of educational time compared to 20 hours in the LB model. We substituted nine hours of podcasts and 10.5 hours of TBL in the TBL/FC experimental group for 12 hours of traditional passive lecture in the LB group. Both groups had the same eight hours of time for a small group simulation.

Table 1 provides a comparison of instructional formats.

**Table 1:** Course format comparison Comparing course format hours for lecture-based control (2012-2014) and experimental (team-based learning/flipped classroom) groups (2015, 2016).

Course model/format	Team-based learning (2015, 2016)/flipped classroom	Lecture based (2012, 2013, 2014)	Same or different?
Small group simulation	8	8	Same
Lecture	0	12	Different +12 for LB
Team-based learning	10.5	0	Different +10.5 for FC/TBL
Podcast recordings	9	0	Different +9 for FC/TBL
Total class time	18.5	20	Different +1.5 for FC/TBL
Total course time	27.5	20	Different +7.5 for FC/TBL

Three to four multiple choice (MC) questions were derived from each 20 to 45-minute digital recording and were placed in a 10-question quiz given at the beginning of each class session, to determine compliance with podcast viewing and encourage engagement. The quizzes were neither scored nor included in our experimental comparison.

The TBL group exercises were 13 cardiac and peri-arrest cases, while the LB model had none. These TBL cases were:

1.       Respiratory distress

2.       Acute coronary syndrome/myocardial infarction (ACS)

3.       Ventricular fibrillation (VF)/cardiac arrest

4.       VF refractory

5.       Care of the post-cardiac arrest patient

6.       Pulseless electrical activity (PEA)

7.       PEA (case 2)

8.       Bradycardia, symptomatic

9.       Asystole

10.    Ventricular tachycardia (VT), stable vital signs, and clinical condition

11.    VT, unstable vital signs, and clinical condition

12.    PSVT (paroxysmal supraventricular tachycardia) with good perfusion

13.    Acute ischemic stroke/administration of tissue plasminogen activator

We managed eight cardiac arrest and peri-arrest simulated cases using high-fidelity mannequins with both teaching formats, with five to nine students and one teacher per group. These were:

1.       ACS from ventricular fibrillation (VF) cardiac arrest, then third-degree atrioventricular block (AVB), then ST elevation myocardial infarction (STEMI)

2.       Atrial fibrillation (AF) with an uncontrolled rapid rate

3.       Stable then progressing to hypotensive/unstable (VT)

4.       PEA

5.       PSVT

6.       Unknown supraventricular rhythm with a rate of 150, either atrial flutter with 2:1 block, or PSVT, or sinus tachycardia

7.       Symptomatic bradycardia

8.       Torsade de pointe (polymorphic VT)

The three written assessments were: 1) a multiple choice (MC) test, 2) a cardiac dysrhythmia test, and 3) a patient management evaluation. The 50-question MC assessment was a standard questionnaire from the AHA, and covered the material in the ACLS Student Manual [14]. This tested basic/advanced airway management, application of algorithms, cardiac arrest pharmacology, and other cases like drowning and acute ischemic stroke management. The passing score for the multiple choice test was determined by the AHA as >84% correct.

The dysrhythmias assessment had 20 brady- and tachyarrhythmias, agonal/asystole, various heart blocks, multifocal atrial tachycardia, and VF, to which the students assigned rhythm diagnoses on a matching basis. The instructors set a passing score for the dysrhythmias assessment of >17/20 rhythms correctly identified.

The case management test was fill-in-the-blanks and included seven clinical situations: ACS, PEA, symptomatic bradycardia, ventricular fibrillation refractory to defibrillation, third-degree heart block, stable and then unstable ventricular tachycardia, and asystole. The instructors set the passing score as >87% correct.

All three written assessments were drawn from material in the ACLS Student Manual or were received from the AHA. Two proficient ACLS instructors who were also experienced clinicians (emergency physician and anesthesiologist, one serving as the regional faculty) developed all testing materials before course implementation. Although we weighted the three parts of written testing alike in the overall “correct answer” score, the maximum test points were 50 for the MC test, 20 for the dysrhythmia test, and 61 for the fill-in-the-blanks clinical scenario test. Cognitive testing was the same for all five classes, both control and experimental, and was three hours in duration.

Statistical methods

We used Stata (Version 14.0, Stata Corp, College Station, TX, USA) to analyze our comparison data. We used the Kruskal-Wallis rank sum test to gauge changes between the two TBL/FC experimental classes and the total scores of three earlier classes delivered in the LB format. We calculated confidence intervals (CIs for differences in proportions using the method of Agresti and Caffo [15]) and considered p < 0.05 as statistically different.

We obtained approval from the local Human Subjects Review Committee.

## Results

A total of 468 students enrolled in the course (259 in the LB format in 2012, 2013, and 2014, and 209 in TBL/FC format in 2015 and 2016). The baseline academic achievement scores for all five classes were the same. The average total Medical College Admissions Test (MCAT) score for the entering classes tested (2015, 2016 versus 2012, 2013, and 2014) were similar at 32.2 versus 31.7, and college grade point average was 3.68 for both LB and TBL/FC classes. 

Two of three tests (MC and fill-in-the-blanks) had statistical increases for the TBL/FC format. For the three tests combined, median scores improved from 93.5% (IQR 90.6, 95.4) to 95.1% (92.5, 96.8, p = 0.0001). For the seven case fill-in-the-blanks test, scores increased from 94.1% for LB (89.6, 97.2) to 94.9% for TBL/FC (91.5, 98.3, p = 0.009). In the 50-question MC, scores increased from 88.0% for LB (84.0, 92.0) to 90.0% for TBL/FC (86.0, 94.0, p = 0.0001). In the 20-question dysrhythmia matching test, students performed well in both learning models with median 100% (p = 0.72). More students did not pass one of three written tests with LB versus TBL/FC (24.7% versus 18.2%) and two or three parts of the written evaluation (8.1% versus 4.3%, respectively, p = 0.01 for change in the number of failed tests). On the contrary, 77.5% of students passed all three parts with TBL/FC versus 67.2% with LB (absolute difference 10.3%, 95% CI 2.2, 18.2%).

Percentage scores with statistical testing for the experimental (TBL/FC) and control (LB) groups are shown in Table 2.

**Table 2:** Percentage scores with statistical testing for the experimental and control groups Test scores (percentage correct, median) for each of three final assessments between the experimental 2015, 2016 TBL/FC model and the control 2012, 2013, and 2014 LB model.

Test modality	Team-based learning/flipped classroom (2015, 2016) (%)	Lecture-based model (2012, 2013, and 2014) (%)	p =
Seven case fill-in-the-blanks clinical scenarios	94.9	94.1	0.009
20 question dysrhythmia matching	100	100	0.72
50 question multiple choice	90.0	88.0	0.0001
% students passing all three tests	77.5	67.2	0.014
Aggregate test scores	95.1	93.5	0.0001

**Figure 1:**
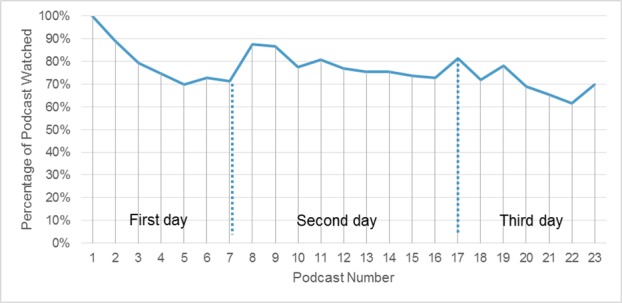
Podcase viewing by students Students (of 209 enrolled) who watched at least some of each of the 23 podcasts for the advanced cardiac life support course for the experimental group (2015, 2016). FC: Flipped Classroom, TBL: Team-Based Learning

We tracked the duration that students in the TBL/FC group watched each instruction video using analytics recorded by the Mediasite Enterprise Video Platform (SonicFoundry, Madison, WI, USA). We used the internet protocol (IP) addresses assigned to students’ devices (desktop or laptop computer, or tablet on a network) to uniquely identify each student. Each of the 23 podcasts was viewed by 160 students on average (range 129-209, 76.6% of 209 students enrolled). The mean duration of the 23 recordings was 23.25 minutes, and the mean proportion viewed per podcast was 74.7%. We determined that there was a general downward trend in the proportion of students viewing at least some part of the assigned podcasts as the TBL phase ran over three days. We also found a downward trend during successive instructional days for the duration of podcast viewing (both shown in Figure 1).

**Figure 2:**
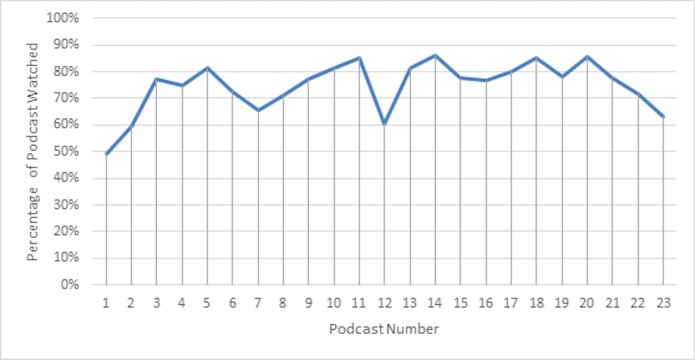
Percentage of duration of podcast recordings watched Percentage of each of the 23 podcast recordings viewed by students who viewed at least some of each podcast (n = 130), for experimental group 2015, 2016. ACLS: Advanced Cardiac Life Support.

We also found that those who viewed the podcasts maintained or increased the percentage of podcast viewed as the course progressed, except for the final podcast on acute ischemic stroke, where the percentage of podcast viewed dropped (Figure 2).

## Discussion

This follow-up work to our previous study [11] confirms our previous findings that the new blended learning model recommended in the 2015 ACLS guidelines [10] should result in an increase in written test scores. This work validates the AHA move to use TBL and FC models for instruction in the 2015 version of the course. Although aggregate scores improved by a small, albeit significant, margin, we found fewer students failing one, two, and three parts of the tests with TBL/FC. Even though the AHA only required the MC test, we wanted to add additional cognitive domain testing to better assess mastery of a broad group of ACLS skills, including dysrhythmia recognition and clinical cardiac arrest and peri-arrest case management.

The TBL/FC ACLS course required 30 hours of preparation (recording of podcasts and worksheet development for TBL) by the trainer in the first year. However, these same videos were used in the class the following year. The AHA has now produced training videos to support the blended learning instructional model for the 2015 guidelines, which would eliminate this extra work for the instructor [10].

The FC model presents information to students prior to class, so that classroom time can be used to foster engagement among students through active learning [16]. This model has recently been more widely applied, though its key elements are drawn from years of learning theory research [17-18]. Only two studies [12-13] have formally assessed electronic learning prior to ACLS classroom instruction, the FC component of our study, and neither found improvement. Our findings, though from a single institution with a relatively large sample, would seem to contradict previous work. We used this FC model in an ACLS course and augmented it with TBL to encourage small group interaction, synthesis of material, and student involvement. We found that TBL, when combined with FC, is an advantageous format for ACLS instruction, as shown by marginal increases in student test scores.

This study had the following limitations. For the FC learning, our tracking analytics reveal that just more than two-thirds of students watched any of the podcasts at all. Those who did viewed on an average 75% of the video duration. This moderates the veracity of any assertion that the new model was an improvement over LB teaching. We scheduled lecture hall time the first half of the day to view that day’s required podcasts, followed by TBL in the afternoons. Other FC course implementations may expect students to watch recordings completely asynchronously. Ultimately, the improved performance on written testing may have resulted from increased total instructional time for the course with the TBL/FC format.

This ACLS class was graded pass or fail, and included a required remediation session per AHA mandate. Eventually, nearly all students passed the ACLS course and achieved certification. This low-stakes learning environment may have decreased study motivation and effort. Furthermore, presenting the ACLS course near the finish of the fourth year, just prior to or after residency match day (variable for each academic year), could have reduced motivation for students to master course material.

## Conclusions

A TBL/FC model for ACLS enhanced written test scores for the fourth-year medical students over the LB format, and significantly lessened the proportion of students who failed one, two, and three parts of the written course evaluation.
